# Interacting with Animals: Understanding their Behaviour and Welfare

**DOI:** 10.1017/awf.2023.26

**Published:** 2023-03-27

**Authors:** Donald M Broom

**Affiliations:** Professor Emeritus, Cambridge University, UK

This book presents many ideas and much useful information. In the Introduction, the authors point out that books and papers about animal behaviour and welfare often ignore early writers and focus on publications in the last ten years. The authors choose to cite the first statements of an idea when explaining relationships between humans and non-humans. They also emphasise that the term animal refers to diverse organisms, from sponges to chimpanzees and humans, and argue that it is not helpful to consider some animals as inferior and some as superior when all are specialised in different ways. They conflict with many recent writers about sentience when they say that animals have an ability to feel. This may be a mis-translation of French in that all animals have some sensory ability and some degree of nervous system function but the capacity to have feelings, i.e. sentience, is currently considered to be limited to vertebrates, cephalopods and decapod crustaceans.

The authors do not subscribe to the view that *Homo sapiens* was especially created through evolution for God’s purpose but consider that, like other animal species, humans emerged by chance. They argue in Chapter 1 that it is wrong to think of non-human animals as mere mechanisms or automata when they are organisms with intrinsic value that are “room-mates” for humans in the world and are deserving of human care and respect. The complex behaviour in many non-human animals was described by Le Roy in 1768 as involving cognition and memory in social and non-social situations. While some writers have assumed that humans are very different from other animals, other authors writing more than a hundred years ago described more similarities than differences and gave examples of sophisticated functioning. In more recent times, observations of behaviour have been of particular importance as evidence for close similarities across animal species, including humans. Examples quoted from the authors’ own work show that farm animals are intellectually sophisticated. It is suggested that it is erroneous to use different words for the same abilities in humans and other species. Aggressiveness, awareness, expectations, fear, pain, suffering and pleasure are not limited to humans and Chapter 2 discusses sensory abilities, many of which are much better in other species than in humans.

Chapter 3 of the book usefully describes social groups and social behaviour. The suggestion that bats in caves may be just a crowd rather than a socially organised group would not apply to the reciprocal altruism that occurs in groups of vampire bats (Desmodontinae). However, the discussion of social behaviour emphasises that tolerance of other group members is frequent and adaptive during many activities, for example, feeding, drinking and migrating. The use of cognitive ability in the everyday life of many species is helpfully explained in Chapter 4 with examples such as those of episodic memory and planning for the future in several mammal and bird species. A mustelid mammal, the grey-headed marten (*Eira barbara*), hides green fruits and then recovers and consumes them when they are ripe while a predatory bird, the black kite (*Milvus migrans*) will move flaming sticks from a fire in order to use them to flush and catch prey. How such abilities and adaptiveness of various non-human species are utilised in adapting to co-existing with humans is the subject of Chapters 5 and 6. Domestication is described as a two-way process with humans and the other species involved both adapting. The responsibilities of humans using other animals are emphasised and welfare is discussed in relation to having an understanding of the needs of members of each species. This approach would be supported by most animal welfare scientists but the definition of *bienêtre* suggested does not make sense in English. As stated by the authors, the concept of welfare must include the positive and the negative. Good treatment (*bien traitance*) of such animals by humans is important but is not in itself welfare because welfare is a characteristic of the individual and good treatment may or may not lead to good welfare. The authors conclude that the use of scientific information, including that following detailed behaviour observation, is vital for assessing welfare.

